# Interactive association between dietary fat and sex on CDH13 cg02263260 methylation

**DOI:** 10.1186/s12920-020-00858-y

**Published:** 2021-01-06

**Authors:** Bei-Hao Shiu, Wen-Yu Lu, Disline Manli Tantoh, Ming-Chih Chou, Oswald Ndi Nfor, Chi-Chou Huang, Yung-Po Liaw

**Affiliations:** 1 Institute of Medicine, Chung Shan Medical University, Taichung City, 40201 Taiwan; 2 Division of Colon-Rectal Surgery, Department of Surgery, Chung Shan Medical University Hospital, Taichung City, 40201 Taiwan; 3 Department of Public Health and Institute of Public Health, Chung Shan Medical University, No. 110 Sec. 1 Jianguo N. Road, Taichung City, 40201 Taiwan; 4 Department of Medical Imaging, Chung Shan Medical University Hospital, Taichung City, 40201 Taiwan; 5 School of Medicine, Chung Shan Medical University, No. 110 Sec. 1 Jianguo N. Road, Taichung City, 40201 Taiwan

**Keywords:** Epigenetics, DNA methylation, Cadherin, cg02263260, Sex, Fat intake

## Abstract

**Background:**

DNA methylation of Cadherin 13 (CDH13), a tumor suppressor gene is associated with gene repression and carcinogenesis. We determined the relation of dietary fat and sex with CDH13 cg02263260 methylation in Taiwanese adults.

**Methods:**

Data of 870 eligible participants (430 men and 440 women) between 30 and 70 years were obtained from the Taiwan Biobank (TWB) database. The association of dietary fat and sex with CDH13 cg02263260 methylation was determined using multiple linear regression.

**Results:**

The association between sex and cg02263260 methylation was significant: beta-coefficient (β) = 0.00532; 95% confidence interval (CI) = 0.00195–0.00868. Moreover, the interaction between sex and dietary fat on cg02263260 methylation was significant (P-value = 0.0145). After stratification by sex, the association of dietary fat with cg02263260 methylation was significant only in women. Specifically, high dietary fat was positively associated with cg02263260 methylation in women (β = 0.00597; 95% CI = 0.00061–0.01133) and the test for trend was significant (P-value = 0.0283).

**Conclusion:**

High fat intake was significantly associated with higher cg02263260 methylation in women and the test for trend was significant. These findings suggest that the association of fat intake and CDH13 cg02263260 might vary by sex and CDH13 cg02263260 methylation levels in women might increase as fat intake increases.

## Background

Dietary habits are among the major modifiable factors for non-communicable diseases (NCDs) like cancer and cardiovascular diseases (CVDs), to mention just a few [1–3]. Unhealthy dietary habits exacerbate the risk of chronic diseases by inducing inflammation and enhancing the production of reactive oxygen species (ROS), which are a driving force for oxidative stress and DNA damage [4–8]. Saturated fats (like cheese, butter, fatty meat, cream, and lard) and unhealthy cooking methods (e.g., frying) increase the risk of colorectal cancer [9–11]. Cancer-causing lifestyle factors influence epigenetic modifications especially DNA methylation [12].

DNA methylation is a well-recognized epigenetic phenomenon characterized by the addition or removal of a methyl group (CH_3_) predominantly at the fifth position of cytosine in a CpG dinucleotide, forming 5-methylcytosine [13–16]. It mediates external effects on gene expressions and plays crucial roles in cellular development, differentiation, and pathogenesis [13–18]. DNA methylation marks possess diagnostic, prognostic, and therapeutic properties that make them potential pathological biomarkers [15]. Promoter methylation, in particular, is regarded as a molecular marker for most human malignancies because different genes exhibit distinct aberrant promoter methylation patterns in several malignancies [19–22].

Cadherin 13 (CDH13), also known as T-cadherin is a tumor suppressor gene located on chromosome 16 [23–26]. Altered methylation profiles and expression of CDH13 could influence oncogenesis [27]. For instance, abnormal CDH13 methylation profiles have been observed in lung [28–33], breast [31, 34–36], cervical [37, 38], colorectal [25, 39–42], pancreatic [43], gastric [44], liver [45], bladder [46], endometrial [46], prostate [47], and nasopharyngeal cancer [39]. CDH13 repression due to aberrant CDH13 promoter methylation has been associated with colorectal [39, 48], NSCLC [32], and pancreatic cancer [43]. However, re-expression of the gene has been associated with suppressed oncogenic processes like proliferation, invasiveness, and angiogenesis [26, 27].

Because CDH13 promoter methylation is common in many human tumors, it has been suggested as an early detection and prognostic marker for human malignancies [27, 46, 49], particularly colorectal [40, 41], breast [34], and non-small cell lung cancer (NSCLC) [29, 32, 33]. The methylation site, cg02263260, located in the promoter region of CDH13 might be important in assessing breast cancer risk and prognosis [49]. CDH13 is a receptor for high-molecular-weight adiponectin [23]. It should be noted that adiponectin is formed and secreted primarily by adipocytes (fat cells) in the adipose tissue [50].

More is yet to be explored in the domain of CDH13 methylation in relation to dietary fat. Moreover, the relationship between CDH13 promoter methylation and sex remains disputable. For instance, in NSCLC, CDH13 promoter methylation and sex were significantly associated [51]. On the contrary, in colorectal [39, 52] and pancreatic cancer [43], they were not significantly associated. Considering that serum adiponectin levels are associated with dietary habits [53], sex [54], and CDH13 [24], we presumed that fat intake and sex may be associated with CDH13 promoter methylation. Therefore, we aimed to determine the association of CDH13 cg02263260 methylation with dietary fat and sex in Taiwanese adults.

## Methods

### Study population and data source

Data, including CDH13 cg02263260 methylation, dietary fat, age, body mass index (BMI), body fat, waist-hip ratio (WHR), exercise, cigarette smoking, alcohol/coffee/tea intake, vegetarian diet, triglycerides (TG), high-density lipoprotein cholesterol (HDL-C), low-density lipoprotein cholesterol (LDL-C), and total cholesterol (TC) were retrieved from the Taiwan Biobank database. The TWB was established in 2005 to ameliorate chronic diseases through the identification of genetic, environmental, and lifestyle disease-causing factors plus the underlying mechanisms [55, 56]. Before enrolment into the TWB project, all volunteers met the eligibility criterion—Taiwanese between 30 and 70 years without a personal history of cancer [57]. All participants signed an informed consent form. Data were collected through questionnaires and physical/biochemical examinations [57]. The TWB dataset contained DNA methylation data of 1142 participants. However, our final sample size was 870 (430 men and 440 women) because we excluded participants who did not complete the questionnaires (n = 3) and those who had missing data (n = 183).

### Dietary fat assessment

Dietary fat was assessed and categorized based on six questions relating to the frequency of consuming fat-rich food over the past month. The questions were: (1) Do you eat meat together with the skin? (2) Do you cook meat/fish with oil? (3) Do you fry vegetables before eating? (4) Do you eat noodles/rice with lard or fried vegetables? (5) Do you fry bean products (tofu, bean curd) before eating? (6) Do you spread cream, butter, or mayonnaise on bread before eating? The responses to these questions were presented on a five-point scale (1 = never, 2 = seldom, 3 = sometimes, 4 = frequently, and 5 = always). We derived the fat-intake score (ranging between 6 and 30) of each participant by summing the responses to all the six questions. Scores were grouped into three: ≤ 12, 13–18, and ≥ 18 corresponding to low, moderate, and high dietary fat, respectively.

### DNA methylation profiling

DNA methylation was determined using pure DNA (i.e., DNA with optical density (OD) 260/280 ratio of 1.6–2.0) that were extracted from whole blood. The EZ DNA Methylation Kit (Zymo Research, CA, USA) was used for bisulfite treatment and conversion of DNA for methylation analysis. DNA methylation profiling was done using the Illumina Infinium MethylationEPIC BeadChip which covers the methylation status of over 850 thousand CpG sites [58]. Quality control of methylation data was performed as previously described [59, 60]. Methylation levels were quantified using beta-values (0–1) and estimated using the formula: M*/*(M + U), with M and U denoting methylated and unmethylated intensity, respectively.

### Covariate assessment

Covariates (age, BMI, body fat, waist-hip ratio, exercise, cigarette smoking, alcohol drinking, coffee/tea intake, vegetarian diet, HDL-C, LDL-C, TG, and TC) were assessed as previously described [59–61]. Smoking and drinking habits were categorized into three groups (never, former and current). Exercise, coffee/tea intake, and vegetarian diet were grouped into two (yes and no). Body fat and waist-hip ratio were categorized as normal or abnormal using cutoff values: 25 and 30% for body fat of men and women, respectively and 0.9 and 0.85 for WHR of men and women, respectively [61].

### Statistical analysis

Data were analyzed with the SAS 9.4 software (SAS Institute, Cary, NC, USA). Differences in basic characteristics of male and female participants were determined with chi-squared (χ^2^) test (for categorical variables e.g., dietary fat, exercise, tea intake, etc.) and t-test (for continuous variables e.g. cg02263260 methylation, age, BMI, etc.). The relationship of dietary fat and sex with CDH13 cg02263260 methylation and the interaction between dietary fat and sex on CDH13 cg02263260 methylation were determined through multiple linear regression analysis and adjustments were made for covariates (age, BMI, exercise, smoking, etc.). The regression results were reported as beta coefficients at 95% CIs. To improve statistical power and reduce type one error, we adjusted for cell-type heterogeneity using the method called Reference-Free Adjustment for Cell-Type composition (ReFACTor) whose details are described elsewhere [62].

## Results

The demographic features of the study participants (n = 870) were stratified by sex as shown in Table 1. The male and female participants constituted 49.43 and 50.57%, respectively. The mean (± SE) ages were 49.36 ± 0.51 years for women and 49.76 ± 0.55 years for men and the methylation levels (beta-values) in men and women were 0.7325 ± 0.0013 and 0.7287 ± 0.0012, respectively. A majority of male and female participants fell in the moderate dietary fat category: 202 men (46.98%) and 205 women (46.59%). The CDH13 cg02263260 methylation levels, dietary fat, body mass index, body fat, cigarette smoking, alcohol/tea intake, vegetarian diet, high-density lipoprotein cholesterol, and triglycerides in men and women were significantly different at P < 0.05 (Table 1).

**Table 1:** Demographic characteristics of participants stratified by sex

Variables	Women	Men	P-value
	(n = 440)	(n = 430)	
CDH13 cg02263260 methylation (beta-value)	0.7287 ± 0.0012	0.7325 ± 0.0013	0.0319
Dietary fat			< .0001
Low (Score ≤ 12)	144 (32.73)	90 (20.93)	
Moderate (Score 13–18)	205 (46.59)	202 (46.98)	
High (Score ≥ 18)	91 (20.68)	138 (32.09)	
Age (years)	49.3614 ± 0.5134	49.7581 ± 0.5479	0.5971
BMI (kg/m^2^)	23.4135 ± 0.1705	25.0735 ± 0.1585	< .0001
Body fat (%)			< .0001
Men < 25; women < 30 (normal)	186 (42.27)	285 (66.28)	
Men ≥ 25; women ≥ 30 (abnormal)	254 (57.73)	145 (33.72)	
Waist-hip ratio			0.7809
Men ≤ 0.9; women ≤ 0.85 (normal)	265 (60.23)	255 (59.30)	
Men > 0.9; women > 0.85 (abnormal)	175 (39.77)	175 (40.70)	
Exercise			0.6095
No	246 (55.91)	233 (54.19)	
Yes	194 (44.09)	197 (45.81)	
Cigarette smoking			< .0001
Never	412 (93.64)	240 (55.81)	
Former	16 (3.64)	102 (23.72)	
Current	12 (2.73)	88 (20.47)	
Alcohol drinking			< .0001
Never	432 (98.18)	352 (81.86)	
Former	3 (0.68)	21 (4.88)	
Current	5 (1.14)	57 (13.26)	
Coffee consumption			0.9589
No	276 (62.73)	269 (62.56)	
Yes	164 (37.27)	161 (37.44)	
Tea consumption			0.0009
No	295 (67.05)	241 (56.05)	
Yes	145 (32.95)	189 (43.95)	
Vegetarian diet			0.0284
No	413 (93.86)	417 (96.98)	
Yes	27 (6.14)	13 (3.02)	
HDL-C (mg/dL)	59.4386 ± 0.6709	49.0023 ± 0.5623	< .0001
LDL-C (mg/dL)	121.1000 ± 1.5480	124.2000 ± 1.5616	0.1568
TG (mg/dL)	106.3000 ± 5.5065	134.1000 ± 4.5752	0.0001
TC (mg/dL)	198.4000 ± 1.6392	194.5000 ± 1.7619	0.1031

n (%) represents categorical data while mean ± standard error (SE) represent continuous data

*BMI* body mass index, *TG* triglycerides, *HDL-C* high-density lipoprotein cholesterol, *LDL-C* low-density lipoprotein cholesterol, *TC* total cholesterol

Table 2 presents the results of multiple linear regression showing the association between dietary fat and CDH13 cg02263260 methylation in all participants. CDH13 cg02263260 methylation was not significantly associated with dietary fat (regardless of the category). Nonetheless, a significant association existed between sex and cg02263260 methylation; with the female sex as the reference group, the male sex was significantly associated with higher CDH13 cg02263260 methylation levels (β = 0.00532, 95% CI = 0.00195–0.00868). Moreover, we found a significant interaction between dietary fat and sex on CDH13 cg02263260 methylation: P-value = 0.0145 (Table 3).

**Table 2:** Multiple linear regression showing the association between dietary fat and cg02263260 methylation in Taiwanese adults

Variables	β	95% CI		P-value
Dietary fat				
Low (reference)	–	–	–	–
Moderate	0.00077	− 0.00246	0.00399	0.6415
High	0.00082	− 0.00309	0.00473	0.6820
Sex				
Women (reference)	–	–	–	–
Men	0.00532	0.00195	0.00868	0.0020
Age	0.00000	− 0.00014	0.00014	0.9874
BMI	− 0.00011	− 0.00064	0.00041	0.6707
Body fat				
Men < 25; women < 30 (reference)	–	–	–	–
Men ≥ 25; women ≥ 30	0.00216	− 0.00135	0.00566	0.2276
Waist-hip ratio				
Men ≤ 0.9; women ≤ 0.85 (reference)	–	–	–	–
Men > 0.9; women > 0.85	− 0.00149	− 0.00442	0.00144	0.3193
Exercise				
No (reference)	–	–	–	–
Yes	0.00106	− 0.00170	0.00381	0.4518
Cigarette smoking				
Never (reference)	–	–	–	–
Former	− 0.00034	− 0.00437	0.00369	0.8676
Current	− 0.00327	− 0.00774	0.00121	0.1524
Alcohol drinking				
Never (reference)	–	–	–	–
Former	0.00054	− 0.00734	0.00842	0.8932
Current	0.00177	− 0.00345	0.00698	0.5062
Coffee consumption				
No (reference)	–	–	–	–
Yes	− 0.00029	− 0.00296	0.00237	0.8293
Tea consumption				
No (reference)	–	–	–	–
Yes	0.00124	− 0.00143	0.00390	0.3618
Vegetarian diet				
No (reference)	–	–	–	–
Yes	0.00105	− 0.00538	0.00748	0.7486
HDL-C	− 0.00007	− 0.00025	0.00011	0.4292
LDL-C	− 0.00001	− 0.00015	0.00013	0.8822
TG	− 0.00001	− 0.00003	0.00002	0.5120
TC	0.00002	− 0.00012	0.00016	0.7621

*BMI* body mass index, *TG* triglycerides, *HDL-C* high-density lipoprotein cholesterol, *LDL-C* low-density lipoprotein cholesterol, *TC* total cholesterol

**Table 3:** Multiple linear regression showing the association between dietary fat and cg02263260 methylation in Taiwanese adults stratified by sex

Variables	Women				Men			
	β	95% CI		P-value	β	95% CI		P-value
Dietary fat								
Low (reference)	–	–	–	–	–	–	–	–
Moderate	0.00285	− 0.00130	0.00700	0.1774	− 0.00213	− 0.00734	0.00307	0.4207
High	0.00597	0.00061	0.01133	0.0292	− 0.00419	− 0.01011	0.00172	0.1642
	P-trend 0.0283				P-trend 0.1600			
Age	− 0.00002	− 0.00022	0.00019	0.8763	0.00002	− 0.00017	0.00022	0.8053
BMI	− 0.00014	− 0.00087	0.00058	0.6969	− 0.00004	− 0.00083	0.00075	0.9285
Body fat								
Men < 25; women < 30 (reference)	–	–	–	–	–	–	–	–
Men ≥ 25; women ≥ 30	0.00247	− 0.00246	0.00739	0.3256	0.00204	− 0.00316	0.00724	0.4413
Waist-hip ratio								
Men ≤ 0.9; women ≤ 0.85 (reference)	–	–	–	–	–	–	–	–
Men > 0.9; women > 0.85	− 0.00139	− 0.00549	0.00270	0.5041	− 0.00173	− 0.00616	0.00271	0.4451
Exercise								
No (reference)	–	–	–	–	–	–	–	–
Yes	0.00332	− 0.00053	0.00716	0.0908	− 0.00095	− 0.00502	0.00311	0.6446
Cigarette smoking								
Never (reference)	–	–	–	–	–	–	–	–
Former	− 0.00148	− 0.01081	0.00784	0.7545	0.00054	− 0.00411	0.00520	0.8192
Current	− 0.00751	− 0.01841	0.00338	0.1759	− 0.00096	− 0.00615	0.00424	0.7171
Alcohol drinking								
Never (reference)	–	–	–	–	–	–	–	–
Former	0.00349	− 0.01757	0.02455	0.7446	− 0.00019	− 0.00911	0.00873	0.9667
Current	0.00571	− 0.01071	0.02213	0.4947	0.00043	− 0.00537	0.00623	0.8842
Coffee consumption								
No (reference)	–	–	–	–	–	–	–	–
Yes	− 0.00125	− 0.00496	0.00247	0.5094	0.00055	− 0.00339	0.00450	0.7828
Tea consumption								
No (reference)	–	–	–	–	–	–	–	–
Yes	0.00350	− 0.00032	0.00731	0.0721	− 0.00092	− 0.00476	0.00293	0.6393
Vegetarian diet								
No (reference)	–	–	–	–	–	–	–	–
Yes	0.00242	− 0.00531	0.01015	0.5393	− 0.00250	− 0.01412	0.00913	0.6732
HDL-C	− 0.00017	− 0.00041	0.00007	0.1718	0.00003	− 0.00025	0.00032	0.8121
LDL-C	− 0.00003	− 0.00022	0.00017	0.7916	− 0.00002	− 0.00024	0.00020	0.8830
TG	− 0.00001	− 0.00005	0.00002	0.3538	0.00000	− 0.00005	0.00004	0.8367
TC	0.00000	− 0.00019	0.00019	0.9953	0.00005	− 0.00017	0.00027	0.6497

Sex*Dietary fat: P-value = 0.0145

*BMI* body mass index, *TG* triglycerides, *HDL-C* high-density lipoprotein cholesterol, *LDL-C* low-density lipoprotein cholesterol, *TC* total cholesterol

In women, moderate dietary fat and CDH13 cg02263260 methylation were not significantly associated: β = 0.00285; 95% CI =  − 0.00130–0.00700. On the contrary, high dietary fat was significantly associated with higher CDH13 cg02263260 methylation levels (β = 0.00597, 95% CI = 0.00061–0.01133). Furthermore, a significant trend (P-value = 0.0283) for fat intake was observed (Table 3). On the contrary, there was no significant association between dietary fat and CDH13 cg02263260 methylation in men (irrespective of dietary fat category). The β; 95% CI was − 0.00213; − 0.00734–0.00307 for moderate and − 0.00419; − 0.01011–0.00172 for high dietary fat. The test for trend was not also significant (Table 3).

After stratification by menopausal status, high dietary fat was positively associated with cg02263260 methylation in non-menopausal women (β = 0.00724; 95% CI = 0.00035–0.01413) and the trend test was significant: P-value = 0.0376 (Additional file 1: Table 1). After adjusting for adiponectin (ADIPOQ) cg16126291 methylation, the association between dietary fat and CDH13 cg02263260 methylation in men and women remained the same (Additional file 1: Tables 2, 3).

## Discussion

In this study on Taiwan Biobank participants, CDH13 cg02263260 methylation was significantly associated with sex but not dietary fat. CDH13 promoter methylation was associated with sex in a study on NSCLC [51]. Conversely, it was not significantly associated with sex in studies on colorectal [39, 52] and pancreatic cancer [43]. Even though CDH13 cg02263260 methylation was not significantly associated with sex in the current study, the interactive association of sex and dietary fat on CDH13 cg02263260 methylation was significant. Stratified analyses yielded significant results only in female participants whose dietary fat was high. That is, compared to low dietary fat, high but not moderate dietary fat was significantly associated with higher levels of CDH13 cg02263260 methylation in women. Notwithstanding, a significant trend for fat intake was observed, inferring that CDH13 cg02263260 methylation levels in women might increase as fat intake increases.

To our knowledge, research on the association between fat intake and CDH13 is lacking and so, there is no available literature to directly compare our findings with. We cannot clearly state why CDH13 cg02263260 methylation and dietary fat intake were not significantly associated in the primary analysis (when partcipants were not stratified). However, the presence of significant results in only women after we stratified participants by sex suggests that sex might play a crucial role in fat-related CDH13 methylation. It is believed that after a high-fat meal, women store relatively high portions of fat [63]. High-fat intake increases the levels of endotoxins in the intestinal mucosa and disrupts the gut microbiota thereby inducing inflammation [8]. Endotoxin-induced proinflammatory responses were found to be greater in women than men and the levels of plasma IL-6 and TNF-alpha in women were significantly higher than in men [64]. The molecular mechanisms underlying the sex differences observed in the current study remain indescribable. It should be noted that after further stratification of the female participants by menopausal status, the association between dietary fat and CDH13 methylation was significant in only non-menopausal women with high fat intake. This implies that the role of hormones especially estrogen cannot be completely disregarded. In osteosarcoma cells, CDH13 expression was modulated by estradiol and progesterone [65]. Moreover, in breast cancer, CDH13 methylation was highly correlated with the down-regulation of estrogen and progesterone receptors [66]. Furthermore, circulating plasma levels of cortisol were significantly increased in women compared to men suggesting more proinflammatory responses to endotoxins in women [64].

CDH13 interacts with adiponectin in the smooth muscle and endothelial cells [24, 67] and high dietary fat reduces adiponectin levels [68]. Low expression of adiponectin is associated with an increased risk of colorectal cancer [69]. Besides, a high intake of fats, especially saturated fats increases the risk of colorectal cancer [9–11]. In our study, we adjusted for ADIPOQ cg16126291, a methylation site in the promoter region of the adiponectin gene which has been validated as being significantly associated with adipogenesis [70]. The observed relationships between fat intake and CDH13 in both men and women were not affected after such adjustments.

Since CDH13 is a tumor suppressor gene, its hypermethylation might result in gene repression as previously shown [27]. However, it was beyond the scope of this study to evaluate the association between CDH13 cg02263260 methylation and gene expression. This is a limitation of our study. Nonetheless, CDH13 down-regulation by aberrant methylation in the CpG island of its promoter is associated with colorectal [39, 48], NSCLC [32], and pancreatic cancer [43]. On the other hand, re-expression of CDH13 in most tumor cell lines inhibits tumor growth through enhanced susceptibility to apoptosis and subdued carcinogenic processes, including invasiveness, proliferation, and angiogenesis [26, 27]. Moreover, the up-regulation of CDH13 brings about an anti-apoptotic effect on the vascular endothelial cells [67] and enhances their migration and proliferation thereby elevating their survival [27]. Due to this, CDH13 is a potential therapeutic target for some cancers [27].

## Conclusions

High fat intake was significantly associated with higher cg02263260 methylation in women and the test for trend was significant. These findings suggest that the association of fat intake with CDH13 cg02263260 might vary by sex and CDH13 cg02263260 methylation levels in women might increase as fat intake increases. As a tumor suppressor gene, CDH13 might repress gene expression when hypermethylated, possibly triggering tumorigenesis.

## Supplementary Information

**Additional file 1:** Association between dietary fat and cg02263260 methylation in Taiwanese adults based on menopausal status and adjustments for ADIPOQ cg16126291 methylation.

## Data Availability

The data that support the findings of this study are available from Taiwan Biobank but restrictions apply to the availability of these data, which were used under license for the current study, and so are not publicly available. Data are however available from Professor Yung Po Liaw (Email address: liawyp@csmu.edu.tw, Tel: + 886424730022 ext. 12102; fax: + 886423248179) upon reasonable request and with permission of Taiwan Biobank.

## Abbreviations

CDH13: cadherin 13
β: beta-coefficient
CI: confidence interval
DNA: deoxyribonucleic acid
NCDs: non-communicable diseases
CVDs: cardiovascular diseases
ROS: reactive oxygen species
CH_3_: methyl group
CpG: cytosine–phosphate–guanine
NSCLC: non-small cell lung cancer
SE: standard error
BMI: body mass index
HDL-C: high-density lipoprotein cholesterol
LDL-C: low-density lipoprotein cholesterol
TG: triglyceride
TC: total cholesterol
TWB: Taiwan Biobank
