# Supplementary material for: Interactive association between dietary fat and sex on CDH13 cg02263260 methylation
Source: BMC Med Genomics. 2021 Jan 6;14:13. doi: 10.1186/s12920-020-00858-y (PMC7788866; doi:10.1186/s12920-020-00858-y)
Supplement: Supplementary file 1 — Additional file 1: Association between dietary fat and cg02263260 methylation in Taiwanese adults based on menopausal status and adjustments for ADIPOQ cg16126291 methylation. [file 12920_2020_858_MOESM1_ESM.docx]

Supplementary Table 1. Multiple linear regression showing the association between dietary fat and cg02263260 methylation in Taiwanese women stratified by menopausal status.

| Variables | Menopause | | | | No menopause | | | |
| --- | --- | --- | --- | --- | --- | --- | --- | --- |
|  | β | 95% CI | | P-value | β | 95% CI | | P-value |
| Dietary fat |  |  |  |  |  |  |  |  |
| Low (reference) | - | - | - | - | - | - | - | - |
| Moderate | 0.00316 | -0.00281 | 0.00913 | 0.2978 | 0.00278 | -0.00321 | 0.00876 | 0.3616 |
| High | 0.00508 | -0.00394 | 0.01409 | 0.2680 | 0.00724 | 0.00035 | 0.01413 | 0.0395 |
|  | P-trend 0.2075 | | | | P-trend 0.0376 | | | |
| Age | -0.00014 | -0.00060 | 0.00033 | 0.5583 | -0.00012 | -0.00052 | 0.00028 | 0.5614 |
| BMI | 0.00053 | -0.00067 | 0.00172 | 0.3856 | -0.00048 | -0.00141 | 0.00045 | 0.3128 |
| Body fat |  |  |  |  |  |  |  |  |
| Men <25; women <30 (reference) | - | - | - | - | - | - | - | - |
| Men ≥25; women ≥30 | 0.00169 | -0.00596 | 0.00935 | 0.6626 | 0.00263 | -0.00395 | 0.00920 | 0.4320 |
| Waist-hip ratio |  |  |  |  |  |  |  |  |
| Men ≤0.9; women ≤0.85 (reference) | - | - | - | - | - | - | - | - |
| Men >0.9; women >0.85 | 0.00268 | -0.00318 | 0.00854 | 0.3687 | -0.00531 | -0.01119 | 0.00056 | 0.0762 |
| Exercise |  |  |  |  |  |  |  |  |
| No (reference) | - | - | - | - | - | - | - | - |
| Yes | 0.00414 | -0.00175 | 0.01003 | 0.1669 | 0.00155 | -0.00372 | 0.00682 | 0.5624 |
| Cigarette smoking |  |  |  |  |  |  |  |  |
| Never (reference) | - | - | - | - | - | - | - | - |
| Former | -0.01027 | -0.02765 | 0.00710 | 0.2449 | 0.00533 | -0.00570 | 0.01635 | 0.3418 |
| Current | -0.00098 | -0.02048 | 0.01853 | 0.9212 | -0.00961 | -0.02266 | 0.00345 | 0.1484 |
| Alcohol drinking |  |  |  |  |  |  |  |  |
| Never (reference) | - | - | - | - | - | - | - | - |
| Former | -0.03242 | -0.07082 | 0.00598 | 0.0975 | 0.02635 | 0.00099 | 0.05171 | 0.0418 |
| Current | 0.02064 | -0.00679 | 0.04807 | 0.1394 | -0.00334 | -0.02372 | 0.01704 | 0.7470 |
| Coffee consumption |  |  |  |  |  |  |  |  |
| No (reference) | - | - | - | - | - | - | - | - |
| Yes | 0.00241 | -0.00344 | 0.00827 | 0.4168 | -0.00466 | -0.00957 | 0.00025 | 0.0625 |
| Tea consumption |  |  |  |  |  |  |  |  |
| No (reference) | - | - | - | - | - | - | - | - |
| Yes | 0.00271 | -0.00310 | 0.00852 | 0.3592 | 0.00385 | -0.00124 | 0.00894 | 0.1370 |
| Vegetarian diet |  |  |  |  |  |  |  |  |
| No (reference) | - | - | - | - | - | - | - | - |
| Yes | 0.00429 | -0.00852 | 0.01711 | 0.5095 | 0.00326 | -0.00649 | 0.01300 | 0.5109 |
| HDL-C | -0.00002 | -0.00038 | 0.00033 | 0.8936 | -0.00045 | -0.00080 | -0.00010 | 0.0131 |
| LDL-C | -0.00004 | -0.00035 | 0.00026 | 0.7747 | -0.00018 | -0.00044 | 0.00008 | 0.1724 |
| TG | -0.00001 | -0.00005 | 0.00004 | 0.6827 | -0.00005 | -0.00011 | 0.00001 | 0.1271 |
| TC | -0.00001 | -0.00030 | 0.00029 | 0.9617 | 0.00018 | -0.00008 | 0.00045 | 0.1749 |
| BMI: body mass index, TG: triglycerides, HDL-C: high-density lipoprotein cholesterol, LDL-C: low-density lipoprotein cholesterol, TC: total cholesterol. | | | | | | | | |

Supplementary Table 2. Multiple linear regression showing the association between dietary fat and cg02263260 methylation in Taiwanese adults (adjusted for ADIPOQ cg16126291).

| Variables | β |  | 95 % CI | | P-value |
| --- | --- | --- | --- | --- | --- |
| Dietary fat |  |  |  |  |  |
| Low (reference) | - |  | - | - | - |
| Moderate | 0.00066 |  | -0.00256 | 0.00388 | 0.6866 |
| High | 0.00077 |  | -0.00313 | 0.00467 | 0.6986 |
| Sex |  |  |  |  |  |
| Women (reference) | - |  | - | - | - |
| Men | 0.00558 |  | 0.00221 | 0.00894 | 0.0012 |
| cg1612629 methylation | 0.11376 |  | 0.02183 | 0.20569 | 0.0153 |
| Age | 0.00000 |  | -0.00014 | 0.00013 | 0.9750 |
| BMI | -0.00008 |  | -0.00061 | 0.00044 | 0.7571 |
| Body fat |  |  |  |  |  |
| Men <25; women <30 (reference) | - |  | - | - | - |
| Men ≥25; women ≥30 | 0.00192 |  | -0.00158 | 0.00542 | 0.2824 |
| Waist-hip ratio |  |  |  |  |  |
| Men ≤0.9; women ≤0.85 (reference) | - |  | - | - | - |
| Men >0.9; women >0.85 | -0.00160 |  | -0.00453 | 0.00132 | 0.2826 |
| Exercise |  |  |  |  |  |
| No (reference) | - |  | - | - | - |
| Yes | 0.00117 |  | -0.00157 | 0.00392 | 0.4019 |
| Cigarette smoking |  |  |  |  |  |
| Never (reference) | - |  | - | - | - |
| Former | -0.00033 |  | -0.00435 | 0.00369 | 0.8719 |
| Current | -0.00315 |  | -0.00761 | 0.00131 | 0.1664 |
| Alcohol drinking |  |  |  |  |  |
| Never (reference) | - |  | - | - | - |
| Former | 0.00029 |  | -0.00758 | 0.00815 | 0.9429 |
| Current | 0.00143 |  | -0.00377 | 0.00664 | 0.5888 |
| Coffee consumption |  |  |  |  |  |
| No (reference) | - |  | - | - | - |
| Yes | -0.00014 |  | -0.00280 | 0.00252 | 0.9184 |
| Tea consumption |  |  |  |  |  |
| No (reference) | - |  | - | - | - |
| Yes | 0.00133 |  | -0.00133 | 0.00399 | 0.3261 |
| Vegetarian diet |  |  |  |  |  |
| No (reference) | - |  | - | - | - |
| Yes | 0.00066 |  | -0.00576 | 0.00708 | 0.8405 |
| HDL-C | -0.00007 |  | -0.00025 | 0.00012 | 0.4749 |
| LDL-C | -0.00001 |  | -0.00015 | 0.00014 | 0.9396 |
| TG | -0.00001 |  | -0.00003 | 0.00002 | 0.5710 |
| TC | 0.00001 |  | -0.00013 | 0.00015 | 0.8462 |
| BMI: body mass index, TG: triglycerides, HDL-C: high-density lipoprotein cholesterol, LDL-C: low-density lipoprotein cholesterol, TC: total cholesterol. | | | | | |

Supplementary Table 3. Multiple linear regression showing the association between dietary fat and cg02263260 methylation in Taiwanese women (adjusted for ADIPOQ cg16126291)

| Variables | Women | | | | Men | | | |
| --- | --- | --- | --- | --- | --- | --- | --- | --- |
|  | β | 95% CI | | P-value | β | 95% CI | | P-value |
| Dietary fat |  |  |  |  |  |  |  |  |
| Low (reference) | - | - | - | - | - | - | - | - |
| Moderate | 0.00281 | -0.00132 | 0.00693 | 0.1818 | -0.00220 | -0.00740 | 0.00301 | 0.4072 |
| High | 0.00585 | 0.00052 | 0.01118 | 0.0316 | -0.00419 | -0.01011 | 0.00172 | 0.1644 |
|  | P-trend 0.0305 | | | | P-trend 0.1616 | | | |
| cg1612629 methylation | 0.15619 | 0.03290 | 0.27949 | 0.0132 | 0.07818 | -0.06559 | 0.22194 | 0.2857 |
| Age | -0.00004 | -0.00024 | 0.00017 | 0.7263 | 0.00003 | -0.00016 | 0.00023 | 0.7469 |
| BMI | -0.00010 | -0.00082 | 0.00062 | 0.7879 | -0.00002 | -0.00081 | 0.00077 | 0.9700 |
| Body fat |  |  |  |  |  |  |  |  |
| Men <25; women <30 (reference) | - | - | - | - | - | - | - | - |
| Men ≥25; women ≥30 | 0.00181 | -0.00311 | 0.00673 | 0.4700 | 0.00213 | -0.00307 | 0.00732 | 0.4221 |
| Waist-hip ratio |  |  |  |  |  |  |  |  |
| Men ≤0.9; women ≤0.85 (reference) | - | - | - | - | - | - | - | - |
| Men >0.9; women >0.85 | -0.00131 | -0.00539 | 0.00276 | 0.5260 | -0.00195 | -0.00640 | 0.00251 | 0.3907 |
| Exercise |  |  |  |  |  |  |  |  |
| No (reference) | - | - | - | - | - | - | - | - |
| Yes | 0.00360 | -0.00023 | 0.00743 | 0.0655 | -0.00094 | -0.00500 | 0.00312 | 0.6497 |
| Cigarette smoking |  |  |  |  |  |  |  |  |
| Never (reference) | - | - | - | - | - | - | - | - |
| Former | -0.00163 | -0.01090 | 0.00765 | 0.7303 | 0.00052 | -0.00413 | 0.00517 | 0.8258 |
| Current | -0.00804 | -0.01887 | 0.00280 | 0.1455 | -0.00085 | -0.00605 | 0.00435 | 0.7478 |
| Alcohol drinking |  |  |  |  |  |  |  |  |
| Never (reference) | - | - | - | - | - | - | - | - |
| Former | -0.00082 | -0.02202 | 0.02038 | 0.9394 | -0.00013 | -0.00905 | 0.00879 | 0.9770 |
| Current | 0.00424 | -0.01213 | 0.02060 | 0.6112 | 0.00024 | -0.00557 | 0.00605 | 0.9353 |
| Coffee consumption |  |  |  |  |  |  |  |  |
| No (reference) | - | - | - | - | - | - | - | - |
| Yes | -0.00114 | -0.00483 | 0.00255 | 0.5446 | 0.00072 | -0.00323 | 0.00468 | 0.7189 |
| Tea consumption |  |  |  |  |  |  |  |  |
| No (reference) | - | - | - | - | - | - | - | - |
| Yes | 0.00344 | -0.00034 | 0.00723 | 0.0747 | -0.00077 | -0.00462 | 0.00308 | 0.6941 |
| Vegetarian diet |  |  |  |  |  |  |  |  |
| No (reference) | - | - | - | - | - | - | - | - |
| Yes | 0.00157 | -0.00614 | 0.00928 | 0.6893 | -0.00248 | -0.01411 | 0.00914 | 0.6748 |
| HDL-C | -0.00018 | -0.00042 | 0.00006 | 0.1404 | 0.00005 | -0.00023 | 0.00034 | 0.7206 |
| LDL-C | -0.00003 | -0.00022 | 0.00016 | 0.7719 | -0.00001 | -0.00023 | 0.00021 | 0.9313 |
| TG | -0.00001 | -0.00005 | 0.00002 | 0.3540 | 0.00000 | -0.00005 | 0.00004 | 0.8908 |
| TC | 0.00000 | -0.00019 | 0.00019 | 0.9933 | 0.00004 | -0.00018 | 0.00026 | 0.7128 |

BMI: body mass index, TG: triglycerides, HDL-C: high-density lipoprotein cholesterol, LDL-C: low-density lipoprotein cholesterol, TC: total cholesterol.

Sex*Dietary fat: P-value = 0.0144
